# A Focus on Astrocyte Contribution to Parkinson’s Disease Etiology

**DOI:** 10.3390/biom12121745

**Published:** 2022-11-24

**Authors:** Giselle Prunell, Silvia Olivera-Bravo

**Affiliations:** 1 Laboratorio de Neurodegeneración y Neuroprotección, Departamento de Neuroquímica, Instituto de Investigaciones Biológicas Clemente Estable, Avenida Italia 3318, Montevideo 11600, Uruguay; 2 Laboratorio de Neurobiología Celular y Molecular, Instituto de Investigaciones Biológicas Clemente Estable, Avenida Italia 3318, Montevideo 11600, Uruguay

**Keywords:** Parkinson’s disease, glial cells, astrocytes, dopaminergic neurons

## Abstract

Parkinson’s disease (PD) is an incurable neurodegenerative disease of high prevalence, characterized by the prominent death of dopaminergic neurons in the substantia nigra pars compacta, which produces dopamine deficiency, leading to classic motor symptoms. Although PD has traditionally been considered as a neuronal cell autonomous pathology, in which the damage of vulnerable neurons is responsible for the disease, growing evidence strongly suggests that astrocytes might have an active role in the neurodegeneration observed. In the present review, we discuss several studies evidencing astrocyte implications in PD, highlighting the consequences of both the loss of normal homeostatic functions and the gain in toxic functions for the wellbeing of dopaminergic neurons. The revised information provides significant evidence that allows astrocytes to be positioned as crucial players in PD etiology, a factor that needs to be taken into account when considering therapeutic targets for the treatment of the disease.

## 1. An Introduction to Parkinson’s Disease

Parkinson’s disease (PD) is the second most common neurodegenerative disease, characterized by motor symptoms that include bradykinesia, muscular rigidity, and resting tremor [1,2]. PD also includes non-motor symptoms, many of them preceding the motor impairment stage for almost a decade, with clinicopathological correlations that are still poorly understood. Here we will focus on the loss of dopaminergic neurons in the substantia nigra pars compacta (SNpc) that is responsible for the motor symptoms and the presence of intraneuronal protein aggregates called Lewy bodies, the hallmarks of PD [1,2].

Aging is the main risk factor for the development of PD, affecting up to 2% of adults over 65 years old, with an incidence increasing 5- to 10-fold between 60 and 90 years of age [3]. A study of the Global Burden of Disease has pointed to neurological disorders as the leading cause of disability between 1990 and 2015, with PD being the fastest growing among them (GBD, 2015). Presently, there is no cure or disease-modifying treatments for PD. Medical management of PD patients is predominantly focused on the restitution of dopamine (DA) levels in the caudate putamen, with levodopa administration being the gold standard treatment [3].

The etiology of PD is poorly understood. Genetic mutations in several genes that result in the development of familiar PD have been identified, but they account for only 5–10% of total cases. Most PD cases are sporadic, and despite decades of intense investigation, the causes of the disease remain mostly unknown [1]. Environmental factors (including some pesticides such as paraquat and rotenone), solvents, metals, and other by-products of industrialization may contribute to the development of PD [4]. On the other hand, a number of lifestyle factors have been associated with reduced risk of developing the disease, including tobacco smoking and coffee intake [5]. It is likely that, in most cases, a complex interplay among predisposing genetic factors, lifestyle habits, and environmental lifetime exposures determine the appearance of the disease.

Studies on animal models and postmortem tissue from PD patients have helped to identify multiple pathways and mechanisms that contribute to the degenerative process of the dopaminergic neurons. Altered proteostasis and, in particular, a dysfunction in α-synuclein (α-syn) degradation, is thought to play a key role in PD. Misfolded and aggregated α-syn is the principal component of Lewy bodies, and point mutations in the α-syn gene or multiplications of the wild type gene cause familiar forms of PD. Furthermore, polymorphisms in the α-syn gene are associated with an increased risk of developing PD [6]. Other PD-linked genes suggesting a dysfunction of the cellular clearance pathways include LRRK2 (leucine-rich repeat serine/threonine protein kinase 2) and ATP132A (ATPase 13A2), which code for proteins related to autophagy and lysosomal metabolism, respectively [7]. In accordance, DA neurons derived from induced pluripotent stem cells from PD patients with LRRK2 mutations showed deficient autophagy mechanisms that might be associated with the accumulation of α-syn [8].

Mitochondrial dysfunction, energetic failure, and oxidative stress (OS) have also been implicated in the pathogenesis of PD. SNpc neurons in PD patients’ present deficits in mitochondrial complex I and mitochondrial dysfunction [9]. Exposure to mitochondrial toxins such as paraquat and rotenone correlates with increased risk of PD [4]. In fact, the inhibitors of mitochondrial complex I, 1-methyl-4-phenyl-1,2,3,6-tetrahydropyridine (MPTP) and rotenone, are widely used to create PD animal models because they induce the degeneration of the dopaminergic neurons of the SNpc [10]. Mutations linked to PINK1 (PTEN-induced putative kinase 1) and parkin genes that codify proteins involved in mitochondrial quality control, are associated with PD [7]. In addition, reactive oxidative species (ROS) production linked to dysfunctional mitochondria can cause widespread oxidative damage to cellular components. In this regard, mutations in the PARK7 gene involving loss of function in the mitochondrial antioxidant DJ-1 protein are related to increased sensitivity to OS and have been proposed as causative of PD [11,12].

Dopaminergic neurons in the SNpc are especially prone to suffer OS. In fact, a high baseline level of OS in the aging SNpc has been observed compared to other neuronal types, including dopaminergic neurons [13]. Large complex unmyelinated axonal arbors together with autonomous pacemaker activity driven by Ca^++^ channels that require substantial amounts of ATP, are aspects involved in the particular vulnerability of DA neurons to OS [14,15]. In addition, dopaminergic neurons are constantly dealing with pro-oxidant products of DA metabolism. Deamination of free DA by monoamine oxidase (MAO) generates H_2_O_2_ that can further participate in the Fenton reaction, generating the highly reactive radical OH, a process that is favored by elevated local levels of iron [16,17,18]. DA is also prone to autoxidation, producing dopamine o-quinone, an unstable molecule that can easily react to form more dangerous molecules, such as aminochrome, that generate ROS, worsening the mitochondrial dysfunction, disturbing the proteasome, and exacerbating endoplasmic reticulum (ER) stress, which, in turn, will induce inflammation and the formation of α-syn oligomers [17]. In accordance, studies on postmortem brains from PD patients indicate increased OS markers and decreased antioxidants defenses [13].

Chronic neuroinflammation is proposed to play a crucial role in PD because of its essential contribution to α-syn aggregation and the neurodegenerative process [19]. Studies on the postmortem tissue of PD patients evidenced markers for glia activation, leucocyte infiltration, and elevated proinflammatory cytokine levels in the SNpc [20,21,22,23,24]. In turn, glial reactivity amplifies and sustains neuroinflammation in a positive feedback loop, where neuroinflammation exacerbates glial proliferation, likely favoring the emergence of highly neurotoxic phenotypes that will release inflammation effectors contributing to perpetuate chronic neuroinflammation and further neurodegeneration [25,26,27,28].

## 2. PD as a Non-Neuronal Cell Autonomous Disease

PD has traditionally been considered as a neuronal cell autonomous pathology in which the sole damage of SNpc dopaminergic neurons suffices to produce the characteristic motor symptoms of the disease. However, growing evidence shows that the damage to key partner cells, as well as to vulnerable neurons, may account for the selective susceptibility of SNpc dopaminergic neurons [29,30,31,32]. Therefore, non-neuronal cell autonomous mechanisms seem to be involved in PD, microglial cells and astrocytes being the most remarkable players.

Gliosis in SN exhibits particular features since this region is remarkably richer in microglial cells compared to other midbrain areas, but is poor in glial fibrillary acidic protein (GFAP) positive astrocytes [24,33]. In the SN of PD patients, exacerbated microgliosis is described, associated with increased ROS and reactive nitrogen species, inducible nitric oxide synthase (iNOS) expression, and the release of proinflammatory prostaglandins, cytokines, and other inflammatory mediators [34]. Tumor necrosis factor-α (TNF-α), interleukin (IL)-1β), IL6, and interferon gamma (IFN-γ), are increased in both SNpc glial cells and in cerebrospinal fluid of PD patients [21,32,35]. These cytokines may amplify and propagate glial reactivity, and consequently, injury to neurons, among other mechanisms, through nuclear factor kappa B (NFkB) activation of the apoptotic machinery [32,34,36]. In fact, this pathway is increased in dopaminergic neurons of PD patients, indicating a role of microglial reactivity during very early PD onset and progression [21]. On the other hand, the involvement of astrocytes in PD initiation is much less studied, although growing evidence suggests a clear but highly complex participation that warrants profound analysis.

## 3. Interplay between Astrocytes and Dopaminergic Neurons under Physiological Conditions

Astrocytes sustain brain homeostasis and provide for CNS defense [28]. They participate in the energetic support to neurons, blood flow regulation, CNS development, synaptogenesis, neurogenesis, synaptic maintenance, as well as in ion and neurotransmitter homeostasis [25,27,28]. Astrocyte roles in brain homeostasis include the exchange of energetic substrates between blood and the brain through different transporters for glucose, lactate/pyruvate, and fatty acids, which provide energetic support to neurons and sustain cellular antioxidant systems [26,28,37]. Astrocyte end-feet, which cover ~95% of the brain capillary surface, are important players in the blood–brain barrier (BBB) and neurovascular unit functions and properties [26,28]. Moreover, they metabolize glucose to lactate, which is later available to neurons [38,39], and it is consensually accepted that this lactate system is linked to glutamate release during neuronal activity. Astrocytes are in charge of glutamate uptake to efficiently end the neurotransmission, which is associated with an increase in Na^+^/K^+^ ATPase activation [25,40]. This causes a significant decrease in ATP levels, raising the rate of glycolysis to produce lactate that could be used by neurons. In addition, the glutamate/glutamine cycle will permit the supply of antioxidants, such as glutathione (GSH) and ascorbate, to neurons, and collaborates with ammonia (NH3) detoxification in order to synthesize glutamine inside astrocytes [41].

**Figure 1:**
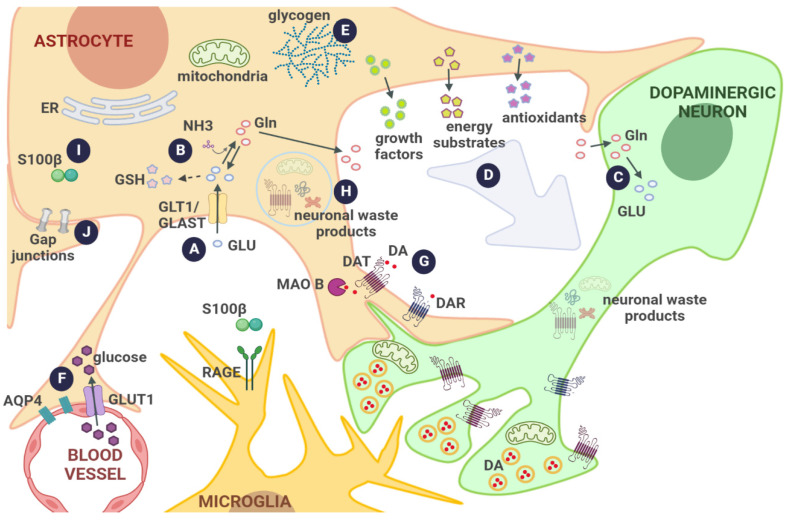
**Normal functions of astrocytes.** (A) Modulation of synaptic activity via GLU transport from the synaptic cleft into the cell; (B) synthesis of Gln and GSH precursors as well as NH_3_ detoxification, all linked to GLU uptake; (C) replenishment of neuronal GLU via the GLU–Gln cycle; (D) release of neurotrophins, energetic intermediates, and antioxidants to provide anaplerotic support to neurons; (E) storage of the main energetic source; (F) transport of glucose from the vasculature, regulation of blood flow by astrocyte end-feet apposing blood vessels, and through the release of vasoactive substances and cell volume regulation mediated by aquaporins; (G) DA activation of DARs and DA uptake mediated by specific transporters and further oxidation by MAOB; (H) normal digestion of neuronal waste products; (I) S100β participation in cytoskeleton stability and calcium signaling; (J) gap junctions that contribute to syncytium formation and coupling, allowing the exchange of small molecules and cell–cell communication. Abbreviations: AQP4, aquaporin; DA, dopamine; DAR, dopamine receptor; DAT, dopamine transporter; ER, endoplasmic reticulum, Gln, glutamine; GLU, glutamate; GLAST, glutamate–aspartate transporter; GLT1, glutamate transporter 1; MAOB, monoamine oxidase B; NH_3_, ammonia; RAGE, receptor for advanced glycosylation end products.

Astrocytes and dopaminergic neurons interact in several ways (Figure 1). For example, astrocytes substantially contribute to CNS monoamine metabolism by taking up extrasynaptic DA through the Na^+^-dependent DA transporter (DAT) [28], which is metabolized by the monoamine oxidase B (MAOB), an enzyme preferentially expressed in astrocytes [42,43]. Astrocytes also express several functional dopamine receptors (DAR), and DA, acting on both D1R and D2R, contributes to induce Ca^++^ transients in astrocytes [44,45,46] and regulates the astrocytic NAD+/NADH redox state [47]. Upon DAR activation, these cells also express and release neurotrophic factors such as the glial-cell-line-derived neurotrophic factor (GDNF) [48,49,50], which is critical for the development and survival of dopaminergic neurons [51]. Fibroblast growth factors (FGF) are other neurotrophins that are site-specific and released by astrocytes. For instance, FGF20 is synthesized at high levels by astrocytes from the SN reticulata but not from the SNpc, and may act on FGF receptors of healthy SNpc dopaminergic neurons [52].

As stated previously, astrocytes provide neurons with the main cellular antioxidant defenses, such as ascorbic acid, metallothioneins (MT)-1 and -2, and cysteine and glutamylcysteine, to synthesize GSH that acts against ROS and pro-oxidant DA quinones produced by DA oxidation [53,54,55,56,57,58]. Interestingly, DA taken up by DAT in astrocytes activates the antioxidant transcription factor Nrf-2, resulting in the upregulation of the expression of several antioxidant proteins, such as MT-1 and -2 and GSH-related enzymes, indicating an interplay among both cell types associated with OS control [56,59,60]. Moreover, the protein DJ-1, which is well recognized as an OS sensor, is highly expressed in astrocytes, and exhibits neuroprotective properties, as suggested by DJ-1 mutations that cause PD and astrocyte PARK7 knockout or -down that results in decreased neuroprotection in PD cellular models [11,61,62]. Astrocytes also contribute to inhibit aminochrome damage to dopaminergic cells by secreting the aminochrome-metabolizing enzyme, controlling neuroinflammatory cascades and the formation of α-syn oligomers [17,63].

In addition, dopaminergic signaling mediated by astrocytic D2R suppresses neuroinflammation by inhibiting the activation of the NLRP3 inflammasome and subsequent cytokine production [64,65,66]. Protective astrocyte actions on SNpc dopaminergic neurons also include the uptake and further degradation of secreted neuronal α-syn and other waste products, such as damaged mitochondria, DAT, and tyrosine hydroxylase (TH), [64,65,66,67,68,69]. In addition, it has been reported that iPSC-derived astrocytes could act as mitochondrial donors to injured dopaminergic neurons, preventing neurodegeneration [70].

**Figure 2:**
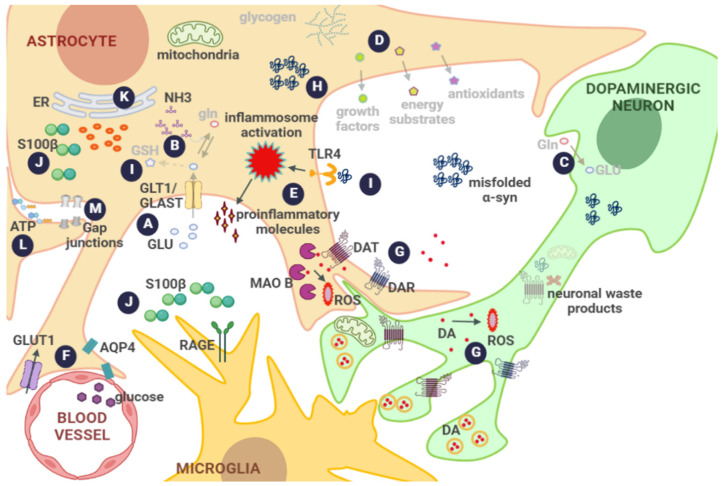
**Potential astrocyte dysfunction in Parkinson’s disease.** (A) Impairment of GLU transporters and increased synaptic GLU and excitotoxicity; (B) defective synthesis of Gln and GSH precursors, impaired NH3 detoxification, and altered cell volume; (C) diminished Gln supply; (D) disturbed anaplerotic support to neurons coexisting with increased release of deleterious soluble mediators (inflammatory cytokines and chemokines); (E) decreased pool of energetic sources; (F) altered glucose transport from the vasculature, decreased coverage of brain capillaries, and release of vasoactive substances, and AQP4 mislocalization resulting in cell volume deregulation; (G) MAOB elevation and increased DA degradation, promoting OS; (H) α-syn intracellular aggregates due to increased uptake and overtaken functions of the proteasome and autophagic pathways; (I) α-syn binding to TLR4 reinforcing inflammasome activation (D), S100β increased expression and extracellular release, disturbing cytoskeleton stability and cell proliferation, and increasing Ca^++^ signaling and activation of RAGE–NFkB-dependent pathways; (J) increased intracellular Ca^++^ eliciting mitochondrial dysfunction and ER stress that will worsen anaplerotic support; (K) increased intracellular protein aggregates could increase the amount of ATP released through hemichannels and via gap junctions, propagating altered calcium signaling and disturbing the glial communication; (L) enhanced hemichannel formation could increase the deleterious signaling to the extracellular medium. All described astrocyte alterations strongly affect the wellbeing of dopaminergic neurons that, per se, exhibit special vulnerability to oxidative and cellular stresses. Abbreviations: AQP4, aquaporin; DA, dopamine; DAR, dopamine receptor; DAT, dopamine transporter; Gln, glutamine; Glu, glutamate; GLAST, glutamate–aspartate transporter; GLT1, glutamate transporter 1; MAOB, monoamine oxidase B; NH_3_, ammonia; RAGE, receptor for advanced glycosylation end products; TLR4, Toll-like receptor 4.

Thus, astrocytes actively contribute to the proper function and survival of dopaminergic neurons in the SNpc through several mechanisms. This also implies that alterations in astrocyte physiology might directly affect dopaminergic neurons, since under pathological conditions, astrocytes fail to maintain homeostatic functions, but also gain functions that might be detrimental to neurons (Figure 2).

## 4. Evidence for Astrocyte Roles in PD as a Non-Neuronal Cell Autonomous Disease

Under damaging conditions, astrocytic responses may include changes in morphology, gene expression, and/or functions [27,37], which is termed astrocyte reactivity, and depends on the context, timing, and type of the injuring stimulus [71]. This complex response usually causes the loss of the main astrocyte homeostatic functions and the gain in toxic properties that may favor scar formation in some cases, the production of proinflammatory cytokines and oxidative species in others, alter glutamate uptake and further impair neurotransmitter synthesis, and reduce anaplerotic support to neurons as well as the defective interplay with the rest of the neural cells. Due to the wide range of defects described in reactive astrocytes, below we will discuss these alterations in relation to dopaminergic neuron damage.

Studies on PD animal models described an intense GFAP+ astrocyte reactivity in both the striatum and the SNpc that parallels dopaminergic neuronal death and remains upregulated even after the main wave of neuronal death has passed [32,72,73]. However, reports on the number and appearance of GFAP+ astrocytes in the degenerating SN from PD patients are conflicting. For instance, some studies described GFAP upregulation and the presence of astrocytes with the typical reactive morphology in the SNpc of PD patients, while others failed to find signs of astrocyte reactivity [24,74,75,76,77]. In any case, astrocytes in the SNpc of PD brains present numerous alterations that can directly affect dopaminergic neurons [30,32,78,79,80,81,82,83,84].

Recently, the use of GFAP as an astrocytic marker in the SNpc has been questioned, raising the possibility that previous reports based on this protein expression have underestimated astrocyte reactivity in PD brains [85]. Other astrocytic markers, such as aquaporin 4 (AQP4) and S100 calcium-binding protein B (S100β), are gaining relevance in PD pathophysiology. AQP4 is the predominant CNS aquaporin that maintains CNS water balance, regulates astrocyte Ca^++^ signal transduction, and participates in the regulation of neurotransmission [86,87,88,89]. PD patients reportedly show lower AQP4 expression [89], and AQP4 deficiency increases the sensitivity of cultured dopaminergic neurons against MPTP/H_2_O_2_ damage, and correlates with cell death and caspase-3 activation [86]. In addition, AQP4 knockout mice were significantly more prone to MPTP-induced neurotoxicity [90], and astrocyte and microglia activation in PD models decreased AQP4 expression [91]. In turn, AQP4 deficiency leads to glial activation in other PD models, and increased inflammatory factors, such as TNF-α and IL-1β, in the midbrain [72,92,93]. Furthermore, it has been proposed that altered astrocytic Ca^++^ signals might cause AQP4 mislocalization and functional deficiency, resulting in neuroinflammation [94]. In turn, alterations in AQP4 probably contribute to increase BBB permeability and alter free water levels in the SNpc of PD patients [95,96].

Increasing evidence also shows the role of S100β+ astrocytes in PD. S100β is widely expressed in astrocytes, in particular, striatal astrocytes, but not in dopaminergic neurons, and is implicated in Ca^++^ homeostasis, energy metabolism, cell proliferation, and cytoskeletal regulation [29,97,98,99]. At nanomolar levels, S100β is neuroprotective, but at micromolar concentrations it is deleterious to neurons, causing Ca^++^ overload, apoptosis, oxidative damage, and excessive neuroinflammation associated with increased ROS production and the release of proinflammatory cytokines [29,97]. S100β can be released to the extracellular medium, and may act as a damage-associated molecular pattern protein through its interaction with the receptor for advanced glycation end products (RAGE), a multiligand receptor that is mainly expressed in neurons and microglia and mediates NFkB-mediated inflammatory responses [29,97]. Both S100β and RAGE levels are increased in postmortem SN of PD patients as well as in MPTP animal models of PD [79,80,99,100]. It also has been observed that astrocytes from 6-hydroxydopamine-treated animals (a widely used PD model) increased S100β secretion in vitro, and S100β increased levels in C6 rat glioma cells positively correlated with the death of cocultured PC12 cells [101,102]. It also has been shown that S100β can significantly contribute to neurodegeneration in S100β-overexpressed PD animal models, and its ablation partially inhibited neurodegeneration [99]. S100β overexpression in transgenic mice induced motor deficits similar to the PD phenotype by suppressing D2R expression, thus likely by affecting DA metabolism and promoting OS [103,104]. In addition, sustained increased S100β levels that may activate RAGE-dependent pathways may induce microglia activation and migration, amplifying neuroinflammation, oxidative damage, and the disturbance of neurotransmitter metabolism, all mechanisms underlying PD pathogenesis [29].

Astrocytic phagocytic processes appear to be altered in PD, likely affecting the efficient clearance of misfolded α-syn, as suggested by abundant deposits of this protein in astrocytes from PD patients and in inducible pluripotent stem cell (iPSC)-derived astrocytes from patients with mutations in LRRK2 [83,84,105,106,107]. Several reports show that α-syn accumulation affects astrocytes in many ways, which include disrupted lysosomes, glutamate transporters, mitochondria and BBB pathways, along with the increased release of proinflammatory cytokines that will negatively affect neuronal survival [108,109,110,111,112,113]. In fact, it was reported that astrocyte α-syn accumulation correlated with the neurodegeneration of cocultured dopaminergic neurons, and the overexpression of α-syn in astrocytes caused gliosis followed by neurodegeneration in rodents [31,68,84,109,110]. Therefore, the inflammatory response in astrocytes elicited by α-syn seems strongly linked to neurodegeneration. In this sense, Toll-like receptors (TLR), in particular TLR4, have been proposed as a connection between PD and neuroinflammation through the immune/neuroinflammatory responses that precede motor and non-motor symptoms [114]. TLR4 is overexpressed in the caudate putamen and in circulating monocytes of PD patients [115]. This receptor is highly expressed in mature human microglial cells and astrocytes under basal conditions [116]. Furthermore, upon exposure to α-syn oligomers, both glial cells release significant amounts of TNF-α in a TLR4-dependent manner [117]. This mechanism has been proposed as a mediator of the alterations caused by α-syn accumulation in the midbrain [114,117]. Moreover, α-syn could bind TLR4-activating inflammatory cascades, which include those dependent on NFkB, c-Jun N-terminal kinase (JNK), and p38 mitogen-activated protein kinases with the downstream overexpression of proinflammatory cytokines, iNOs and COX2, involved in cell degeneration [114,118,119]. Astrocytes also express TLR3, the signaling of which also may initiate the neuroinflammatory response [120]. In addition, activation of the dopaminergic D3R, which is selectively expressed in dopaminergic neurons and astrocytes but not in microglial cells, in the SN and ventral tegmental area in PD, occurs under inflammatory conditions and sustains neuroinflammation, causing a positive feedback loop [64,121].

Excitotoxicity resulting from dysfunctional astrocytic glutamate transporters also appears to contribute to PD pathology [122]. For instance, a reduction in glutamate uptake and in the expression of glutamate transporter-1 (GLT1) and glutamate/aspartate transporter (GLAST) in the nigrostriatal pathway have been described in different PD rodent models [122,123,124]. Furthermore, studies conducted by Zhang et al. show that GLT1 deficiency in the SNpc of mice induces motor deficits and dopaminergic neuronal death associated with astroglia and microglia reactivity [122]. In this regard, it has been suggested that increased D2R stimulation due to a surge of DA in the early PD stage may result in an aberrant astrocytic Ca^++^ signal that will downregulate GLT1 expression, facilitating excitotoxic damage [46,94]. In accordance, a nuclear magnetic resonance study in PD patients reported increased glutamate levels in the putamen ipsilateral to the more affected hemibody [125]. Altered astrocytic glutamatergic metabolism will also compromise NH_3_ detoxification and GSH synthesis, with the consequent affectation of metabolic and antioxidant support to neurons [124].

In addition, neurovascular decoupling that impairs the upregulation of glucose transporter 1 (GLUT1) and glycolysis in astrocytes, which under physiological conditions reinforce the supply of activated neurons, has been described in some PD patients [126,127]. This impaired astrocyte response in PD may be partially explained by a limited functional expression of GLUT1, which is recognized as the master controller of neuronal glucose utilization, resulting in a decreased pool of energetic sources such as glycogen available to neurons, thus providing a link between neurodegenerative disorders and energy metabolism [37].

Another astrocytic protein that has been associated with PD is MAOB, the levels of which are significantly increased in the SNpc astrocytes in PD [128,129,130]. Increased MAOB has been proposed to contribute to disease pathogenesis by an increased degradation of DA and other substrates, which results in H_2_O_2_ overproduction and subsequent OS [17]. Recently, it has also been suggested that increased MAOB activity in astrocytes potentiate the astrocytic synthesis and release of gamma aminobutyric acid (GABA) [128,129,131]. Increased extracellular GABA activates extrasynaptic GABAA receptors that inhibit the neighboring dopaminergic neuronal activity, leading to a substantial decrease in TH with the consequent deficiency of DA, which can lead to parkinsonian motor symptoms [128,131,132]. In accordance, MAOB inhibitors have protective effects on PD, preventing dopaminergic neuron degeneration and decreasing parkinsonian symptoms, especially when applied to early phase patients [133]. MAOB genetic ablation or silencing also alleviated parkinsonian motor symptoms [128,129].

Astrocytes also possess a plethora of spontaneous Ca^++^ signals that regulate diverse signaling pathways that could act in an autocrine manner to modulate nearby cells [134,135]. In addition, increased levels of Ca^++^ ER and altered mitochondrial functions are described in PD patients, which, in turn, will worsen the redox status, thus impairing the antioxidant support and lactate shuttle to neurons. In accordance, Ca^++^ channel blockers have been proposed to treat PD [136].

Finally, enhanced hemichannel formation could increase astrocyte-derived deleterious signaling to the extracellular medium in PD. In this sense, in the MPTP model, astrocytic connexin-43 (Cx43) hemichannel permeability was increased and accompanied by elevated intracellular Ca^++^ levels in midbrain slices, while the administration of a hemichannel inhibitor avoided dopaminergic neuronal loss and inhibited microglial activation [137]. On the other hand, rotenone administration in vivo or in vitro increases Cx43 protein level and phosphorylation in astrocytes [138]. Furthermore, α-syn enhances the opening of Cx43 and Pannexin 1 hemichannels in mouse cortical astrocytes, resulting in altered intracellular Ca^++^ dynamics, nitric oxide production, and gliotransmitter release, including ATP [139]. Released ATP could act in a paracrine fashion by activating purine receptors on adjacent astrocytes, increasing the ATP release and intracellular Ca^++^ mobilization through a feed-forward mechanism that could alter glial cell communication further [25]. Therefore, astrocytes in the SNpc of PD brains and in PD models present numerous alterations that can directly or indirectly affect the survival of dopaminergic neurons.

## 5. Conclusions

Astrocytes participate in almost all CNS functions, and play significant roles in the initiation and progression of neurodegenerative diseases, including PD. Here we review the mounting evidence that supports a considerable interplay between astrocytes and dopaminergic neurons, as well as the impact of astrocyte dysfunction on the survival of these neurons, mostly in view of their specialized requirements and the low astrocytes/neuron ratio in the SN, which could imply more critical effects than in other parts of the brain. Despite great strides in understanding how neurons and glial cells act together, and how disease disrupts these interactions, there is still a long way to go to fully elucidate the non-neuronal cell autonomous mechanisms involved in PD; such information is relevant when considering the assessment of novel therapeutics for this disease.
